# The accessory parotid gland and facial process of the parotid gland on computed tomography

**DOI:** 10.1371/journal.pone.0184633

**Published:** 2017-09-15

**Authors:** Dongbin Ahn, Chang Ki Yeo, Soon Yong Han, Jeong Kyu Kim

**Affiliations:** 1 Department of Otolaryngology-Head and Neck Surgery, School of Medicine, Kyungpook National University, Daegu, Korea; 2 Department of Otolaryngology-Head and Neck Surgery, School of Medicine, Keimyung University, Daegu, Korea; 3 Department of Otolaryngology-Head and Neck Surgery, School of Medicine, Catholic University of Daegu, Daegu, Korea; The Chinese University of Hong Kong, HONG KONG

## Abstract

The purpose of this study was to determine the incidence of an anterior extension of the parotid gland, such as an accessory parotid gland (APG) or facial process (FP) and to evaluate its characteristics on computed tomography (CT) scans. We reviewed CT scans of 1,600 parotid glands from 800 patients. An APG on CT was defined as a soft-tissue mass of the same density as the main parotid gland, located at the anterior part of the main parotid gland, and completely separate from the main parotid gland. An FP was defined as a lobe of the parotid gland protruding anteriorly over the anterior edge of the ramus of the mandible on CT and showing continuity with the main gland. The overall incidence rates and characteristics of APGs and FPs were evaluated according to age, sex, and side. The incidence rates of APGs and FPs were 10.2% (163/1,600) and 28.3% (452/1,600), respectively. The mean size of an APG was 15.8 mm × 5.0 mm and the mean distance from the main parotid gland was 10.5 mm. The FP reached anteriorly between the anterior edge of the mandibular ramus and the anterior border of the masseter muscle in 405 (89.6%) cases, while it extended over the anterior border of the masseter muscle in 47 (10.4%) cases. The incidence rates of APGs and FPs decreased and increased, respectively, with increasing age, showing significant linear correlations. However, the incidence of an anterior extension of the parotid gland (either an APG or an FP) was similar across all age groups. The present study showed that CT might be helpful in identifying anterior extensions of the parotid gland including APGs and FPs. The anatomical information gained from this study contributes to a better understanding of APGs and FPs and how their incidence changes with age.

## Introduction

An accessory parotid gland (APG) is a variation of an anterior extension of the parotid gland found in 21–69% of cadaveric cases [1–4]. An APG is defined as a collection of salivary tissue separate from the main parotid gland that usually ranges in size from a pea to a kidney bean, is located 0.6 cm anterior from the anterior edge of the main parotid gland on average, and has a flattened appearance resulting from compression between the masseter muscle and skin [1, 3]. However, the farthest reported APG was found on the buccal fat pad at the anterior border of the masseter muscle, indicating a great variation in anatomical location [1]. Another type of anterior extension of the parotid gland is a facial process (FP) of the parotid gland, defined as the parotid lobe protruding anteriorly along the course of the parotid duct while maintaining continuity with the main gland [1, 4]. The clinical significance of these anatomical features was recognized after multiple issues were noted. First, a complete parotidectomy can be hampered by missing an APG or FP. Second, clinical suspicion and awareness of APGs and FPs are important to establish appropriate management plans for mid-cheek masses, and to differentiate them from other soft-tissue masses, such as an epidermoid cyst, lipoma, haemangioma, and lymphangioma [3, 5, 6]. Third, the histological characteristics of APGs and FPs can differ from those of the main parotid gland, such that tumours arising from an APG or FP can show different biological behaviours from tumours of the main parotid gland. Although tumours arising from an APG represent only 1–8% of all parotid gland tumours, 26–52% of all APG tumours are malignant, which is a much higher rate compared to the < 20% malignancy rate for tumours of the main parotid gland [3, 7].

Several studies have evaluated APG tumours or mid-cheek masses by using computed tomography (CT) imaging [5, 6]. However, no study has identified APGs or FPs as normal anatomical variations of the parotid gland in non-tumour patients by using CT imaging. For this reason, the current knowledge on APGs and FPs still relies on a small number of fundamental cadaveric studies that have been performed several decades ago [1, 4, 8, 9]. In current clinical circumstances, large amount of data is available from various imaging modalities that can reflect actual anatomical structures and can provide additional information complementing previous cadaveric studies. Therefore, in this study, we further investigated the incidence rates and characteristics of APGs and FPs based on a large number of anatomical imaging data sets.

## Materials and methods

### Patients

This study was approved by the institutional review boards of the involved institutions (registration number 201501021; Kyungpook National University Hospital, Keimyung University Hospital, and Daegu Catholic University Medical Center) and was performed as a retrospective review of medical records. The institutional review boards waived the need for informed consent for this retrospective study, which complied with the Health Insurance Portability and Accountability Act. From January 2010 to June 2014, we reviewed 1,600 parotid gland sides from 800 patients who had undergone neck CT scans to evaluate symptoms or signs in the neck other than those of the salivary gland or mid-cheek region. Patients were selected randomly using a stratified strategy according to sex and age. The sex ratio was matched at 1:1. The patients were divided into eight age groups by decade from the first decade to the eighth decade, and each age group included 200 parotid sides from 100 patients. Patients who had a history of radiotherapy of the head and neck region, disease possibly related to the salivary glands including an APG tumour or mid-cheek mass, symptoms of dry mouth, and metallic artefacts that could disturb an accurate imaging analysis were excluded.

### Image interpretation

**Fig 1:**
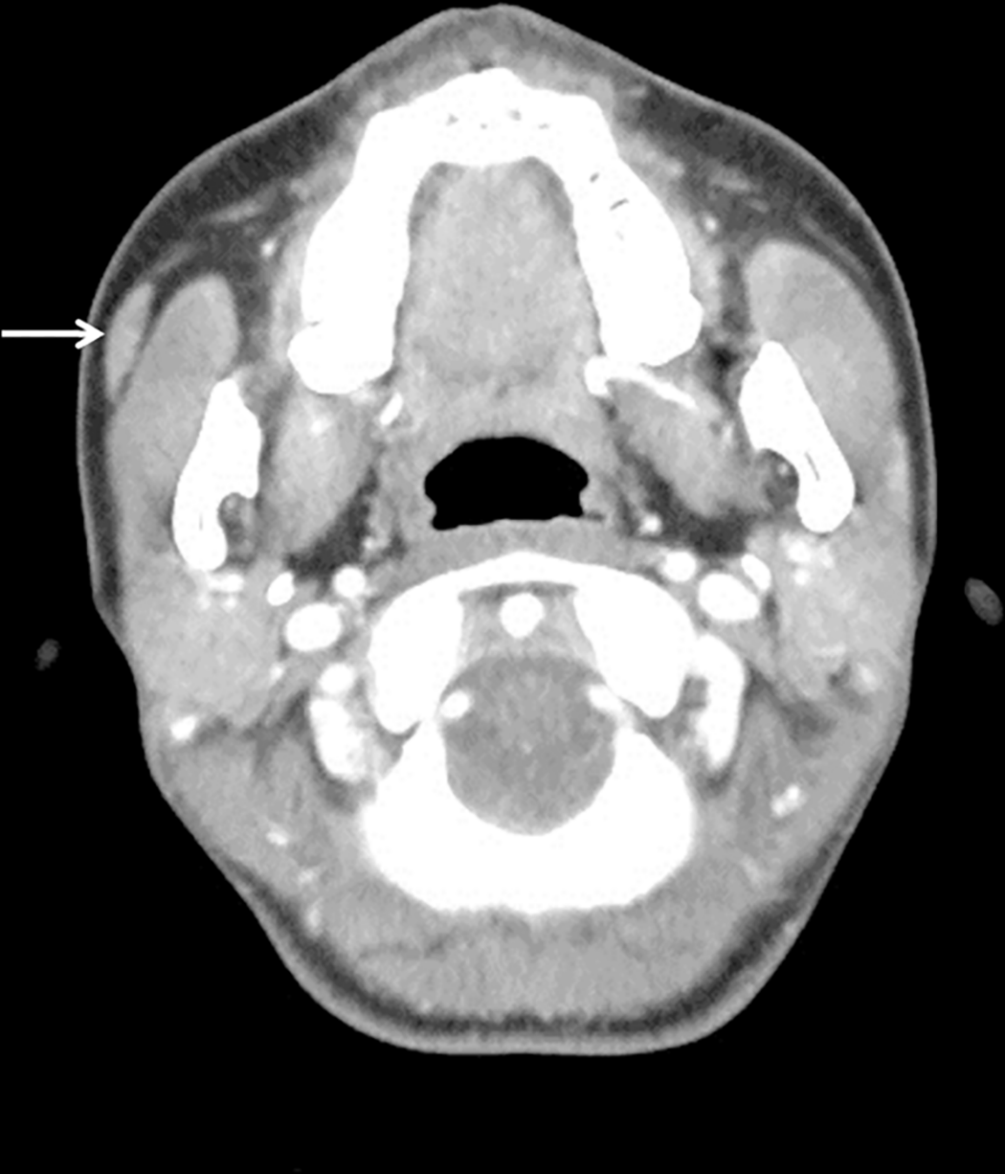
A representative example of an accessory parotid gland. A soft-tissue mass that has the same density as the main parotid gland is shown, is located at the anterior part of the main parotid gland, and is completely separate from the main parotid gland on every slice of the CT scan (arrow).

**Fig 2:**
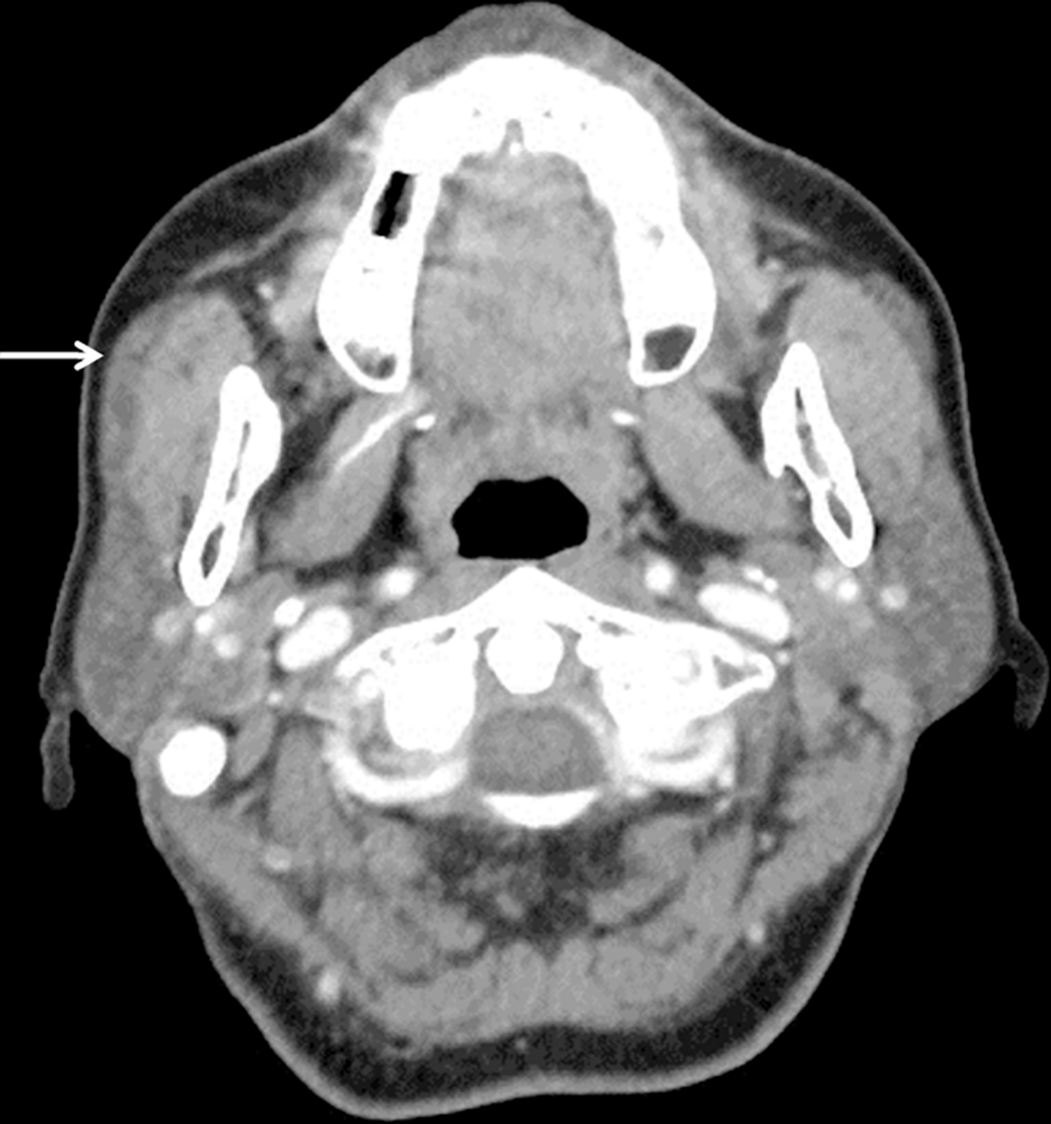
A representative example of a facial process of the parotid gland. Protrusion of the parotid gland anteriorly over the anterior edge of the ramus of the mandible and continuity with the main gland distinguishes a facial process of the parotid from an accessory parotid gland (arrow).

All CT scans were obtained on a HiSpeed or LightSpeed scanner at a slice thickness of 3 mm after the administration of 125 mL of intravenous contrast material. Images were viewed on a picture archiving and communication system (PACS) workstation in axial stack mode without reconstruction. All measurements were obtained with measurement tools included on the PACS workstation. Four experienced head and neck surgeons and one radiologist participated in the review of the CT scans. An APG on CT was defined as a soft-tissue mass of the same density as the main parotid gland, located at the anterior part of the main parotid gland, and completely separate from the main parotid gland in every axial slice of the CT scan (Fig 1). An FP of the parotid gland, which was distinguished from an APG, was defined as a lobe of the parotid gland protruding anteriorly over the anterior edge of the ramus of the mandible on CT and showing continuity with the main gland (Fig 2).

To ensure that APGs and FPs had the same density as the main parotid gland, we quantitatively measured Hounsfield units of suspicious APGs and FPs and the main parotid gland. First, using a PACS workstation, we selected a region of interest (ROI) within the main parotid gland on the axial CT scan to measure Hounsfield units of the main parotid gland. Then, mean Hounsfield units and the corresponding minimum, maximum, and standard deviation values for the ROI were calculated automatically. Next, we measured Hounsfield units of suspicious APGs and FPs using the same methods and compared the values with those of the main parotid gland. A suspicious APG or FP was considered real if the mean Hounsfield units of the suspicious APG or FP were within the range of mean Hounsfield units ± standard deviation of the main parotid gland.

### Assessment parameters

The overall incidence rates of APGs and FPs and differences according to age, sex, and side were evaluated. With regard to age, we compared the incidence rates of APGs and FPs between eight age groups. Pearson’s correlation analysis was performed to evaluate age-related changes in the incidence rates of APG and FP. In each case with an APG, its size and distance from the main parotid gland were also measured; the longest and shortest diameter of the APG was measured on a × 3.0-magnified image for precise measurements in PACS. Regarding the distance from the main parotid gland, the shortest direct distance between the anterior border of the main parotid gland and the posterior border of the APG was measured on a × 3.0-magnified image. SPSS for Windows (version 12.0; SPSS Inc., Chicago, IL, USA) was used to analyse the data. Continuous data are presented as the mean and range and were compared between the groups using the independent Student’s *t* test. To test for differences between groups of categorical variables, the chi-squared test or Fisher’s exact test was used. Statistical significance was defined as *p* < 0.05, and all *p*-values are two-sided.

## Results

### Incidence rates and characteristics of accessory parotid glands

Among 1,600 parotid glands from 800 patients, APGs were found on 163 parotid sides with an overall incidence of 10.2% (163/1,600). The incidence *rate*s of APGs in the male and female sexes were 9.4% (75/800) and 11.0% (88/800), respectively, and the difference was not statistically significant (*p* = 0.283). The incidence rates of APGs on the right and left parotid sides were 10.4% (83/800) and 10.0% (80/800), respectively, and the difference was not statistically significant (*p* = 0.804). In 23 (2.9%) out of 800 patients, APGs were found on both sides. The mean size of an APG was 15.8 ± 4.8 mm (range, 3.8–29.4 mm) × 5.0 ± 1.4 mm (range, 2.0–10.4 mm). The mean distance from the APG to the main parotid gland was 10.5 ± 6.1 mm (range, 1.0–37.0 mm).The mean ages of patients with and without APGs were 31.8 ± 21.7 years (range, 2–79 years) and 40.7 ± 22.6 years (range, 0–79 years), respectively, and the difference was statistically significant (*p* < 0.001) (Table 1).

**Table 1:** Demographics according to the presence of an accessory parotid gland.

Variables	APG (+) (n = 163)	APG (-) (n = 1,437)	*p*-value
Age (years)	31.8 ± 21.7 (range, 2–79)	40.7 ± 22.6 (range, 0–79)	<0.001
Sex			
Men (n = 800)	75 (9.4%)	725 (90.6%)	0.283
Women (n = 800)	88 (11.0%)	712 (89.0%)	
Side			
Right (n = 800)	83 (10.4%)	717 (89.6%)	0.804
Left (n = 800)	80 (10.0%)	720 (90.0%)	
Size of APG			
Longest diameter (mm)	15.8 ± 4.8 (range, 3.8–29.4)	-	-
Shortest diameter (mm)	5.0 ± 1.4 (range, 2.0–10.4)	-	
Distance of APG from the main parotid gland (mm)	10.5 ± 6.1 (range, 1.0–37.0)	-	-

APG, accessory parotid gland

Continuous data are presented as the mean (range).

**Fig 3:**
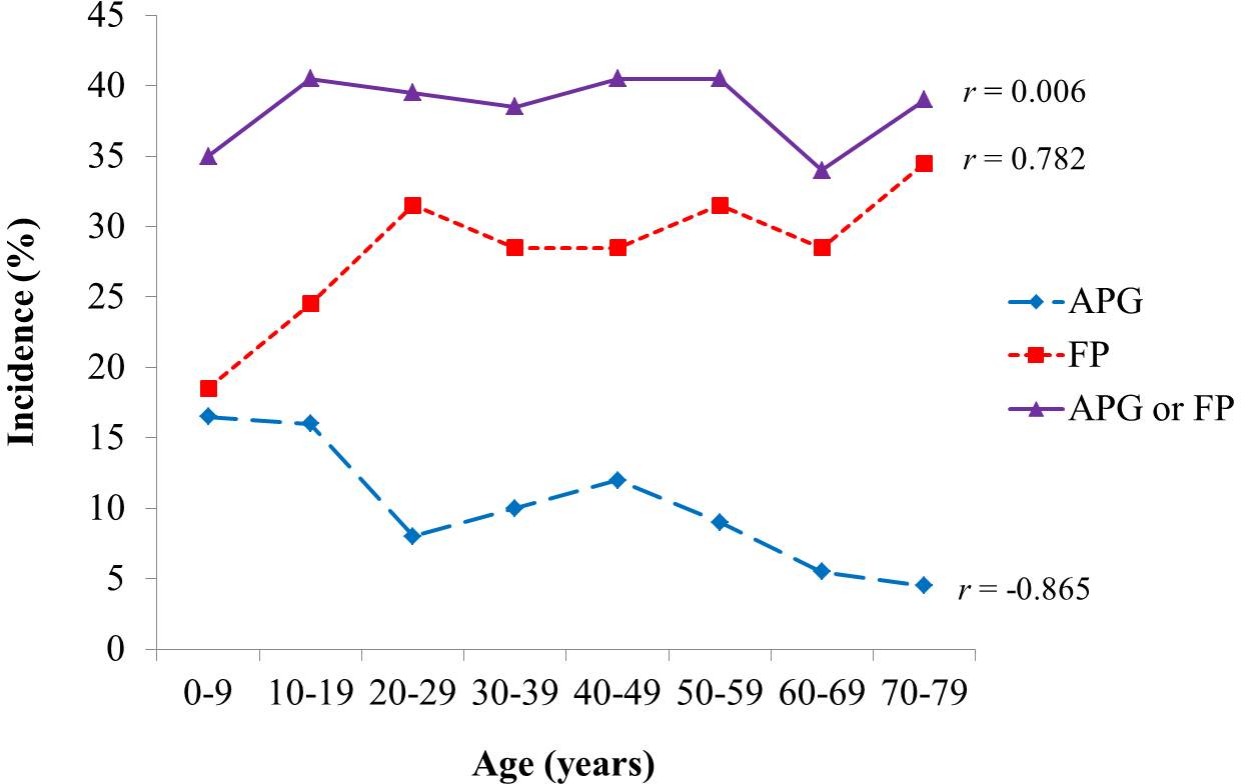
Changes in the incidence rates of an accessory parotid gland (APG), facial process (FP), and either, according to age.

The incidence of APGs by age group was 16.5% (33/200) in the first decade (0–9 years old), 16.0% (32/200) in the second decade (10–19 years old), 8.0% (16/200) in the third decade (20–29 years old), 10.0% (20/200) in the fourth decade (30–39 years old), 12.0% (24/200) in the fifth decade (40–49 years old), 9.0% (18/200) in the sixth decade (50–59 years old), 5.5% (11/200) in the seventh decade (60–69 years old), and 4.5% (9/200) in the eighth decade (70–79 years old). Pearson’s correlation coefficient (r) for the incidence of an APG according to the age groups was -0.865 with *p* = 0.006, indicating that there was a significant negative linear relationship between the age and incidence of an APG (Fig 3).

### Incidence of facial processes

FPs of the parotid gland were found on 452 parotid sides with an overall incidence of 28.3% (452/1,600). The incidence rates of FPs in the male and female sexes were 31.4% (251/800) and 25.1% (201/800), respectively, and the difference was statistically significant (*p* = 0.005). The incidence rates of FPs on the right and left parotid sides were 30.8% (246/800) and 25.8% (206/800), respectively, and the difference was statistically significant (*p* = 0.026). In 152 (19.0%) out of 800 patients, FPs were found on both sides. Among a total of 452 cases with FPs, the anterior end of the FP reached between the anterior edge of the mandibular ramus and the anterior border of the masseter muscle in 405 (89.6%) cases, while it extended over the anterior border of the masseter muscle in 47 (10.4%) cases. The mean ages of patients with and without FPs were 42.7 ± 22.1 years (range, 2–79 years) and 38.6 ± 22.7 years (range, 0–79 years), respectively, and the difference was statistically significant (*p* = 0.001) (Table 2).

**Table 2:** Demographics according to the presence of a facial process of the parotid gland.

Variables	FP (+) (n = 452)	FP (-) (n = 1,148)	*p*-value
Age (years)	42.7 ± 22.1 (range, 2–79)	38.6 ± 22.7 (range, 0–79)	0.001
Sex			
Men (n = 800)	251 (31.4%)	549 (68.6%)	0.005
Women (n = 800)	201 (25.1%)	599 (74.9%)	
Side			
Right (n = 800)	246 (30.8%)	554 (69.2%)	0.026
Left (n = 800)	206 (25.8%)	594 (74.2%)	

FP, facial process

Continuous data are presented as the mean (range).

The incidence of FPs by age group was 18.5% (37/200) in the first decade (0–9 years old), 24.5% (49/200) in the second decade (10–19 years old), 31.5% (63/200) in the third decade (20–29 years old), 28.5% (57/200) in the fourth decade (30–39 years old), 28.5% (57/200) in the fifth decade (40–49 years old), 31.5% (63/200) in the sixth decade (50–59 years old), 28.5% (57/200) in the seventh decade (60–69 years old), and 34.5% (69/200) in the eighth decade (70–79 years old). Pearson’s correlation coefficient (r) for the incidence of an FP according the age groups was 0.782 with *p* = 0.022, indicating that there was a significant positive linear relationship between age and the incidence of an FP (Fig 3).

### Incidence of an anterior extension of the parotid gland

The overall incidence of an anterior extension of the parotid gland (either an APG or FP) was 38.4% (615/1,600). The incidence rates of the anterior extension in the male and female sexes were 40.8% (326/800) and 36.1% (289/800), respectively, and the difference was not statistically significant (*p* = 0.057). The incidence rates of the anterior extension on the right and left parotid sides were 41.1% (329/800) and 35.8% (286/800), respectively, and the difference was statistically significant (*p* = 0.027). The mean ages of patients with and without anterior extension of the parotid gland were 39.8 ± 22.5 years (range, 0–79 years) and 39.7 ± 22.7 years (range, 2–79 years), respectively, and the difference was not statistically significant (*p* = 0.935) (Table 3).

**Table 3:** Demographics according to presence of an anterior extension of the parotid gland (either an accessory parotid gland or facial process).

Variables	Anterior extension of the parotid gland (+) (n = 615)	Anterior extension of the parotid gland (-) (n = 985)	*p*-value
Age (years)	39.8 ± 22.5 (range, 0–79)	39.7 ± 22.7 (range, 2–79)	0.935
Sex			
Men (n = 800)	326 (40.8%)	474 (59.2%)	0.057
Women (n = 800)	289 (36.1%)	511 (63.9%)	
Side			
Right (n = 800)	329 (41.1%)	471 (58.9%)	0.027
Left (n = 800)	286 (35.8%)	514 (64.2%)	

Continuous data are presented as the mean (range).

Pearson’s correlation coefficient (r) for the incidence of an anterior extension of the parotid gland according to the age groups was 0.006 with *p* = 0.989, indicating that there was no positive or negative relationship between age and the incidence of an anterior extension of the parotid gland (Fig 3).

## Discussion

The present study showed that CT might be helpful in identifying normal anatomical variations of the parotid gland including APGs and FPs. Previous imaging studies on APGs have focused primarily on tumours arising from an APG or mid-cheek mass rather than on the identification of anterior extensions of the parotid gland (APGs and FPs) as normal anatomical variations.

**Fig 4:**
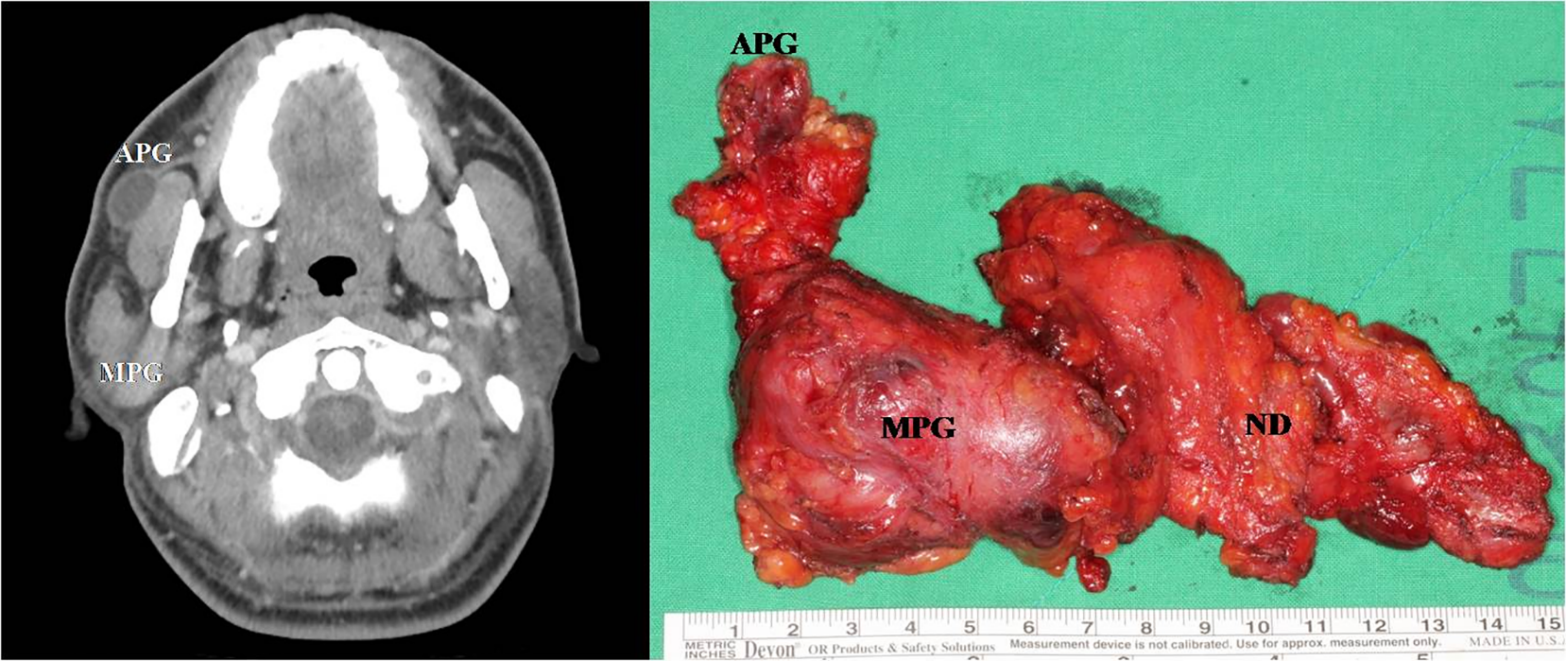
Computed tomography (left) and a surgical specimen (right) show tumours involving the right main and accessory parotid glands separately with no continuity between the two lesions. The final histopathology of the tumours demonstrated acinic cell carcinomas in both the main and accessory parotid glands, without continuity in the salivary tissue between them. Abbreviations: APG, accessory parotid gland; MP, main parotid gland; ND, neck dissection.

In the present study, the incidence of an APG was 10.2% among 1,600 parotid sides on CT. This is a relatively low incidence compared with those of previous cadaveric studies, which reported incidence rates ranging from 21%–69% [1, 4, 8]. This is possibly because the CT was scanned at a slice thickness of 3 mm, and small APGs might have been missed. In addition, an FP of the parotid gland was found in 28.3% of 1,600 parotid sides, and an anterior extension of the parotid gland, either an APG or an FP, was found in 38.4% of the cases. To the best of our knowledge, this is the first report to identify APGs and FPs of the parotid gland on CT, and the information gained form this study may have clinical implications with regard to complete removal of the gland in the parotid surgery and setting appropriate irradiation field in radiation therapy. Although an APG is completely detached from the main parotid gland, it usually lies on or above the parotid duct and has one or more tributaries emptying into the major parotid duct, Stensen’s duct [4]. Therefore, we can assume that an APG is indirectly connected to the main parotid gland through the path of the parotid duct, and a failure to remove or irradiate an APG may be associated with tumour recurrence. In fact, we have experienced a case of parotid cancer involving the main parotid gland and a separate APG with the same histology but no continuous lesion present between the tumours in the main gland and the APG on a CT scan or in surgical specimens (Fig 4). The final histopathology of the tumour demonstrated acinic cell carcinoma in both the main parotid gland and the APG, without continuity in the salivary tissue between them. We believe that this case shows that tumour cells from the main gland can be transplanted or metastasized to the APG or vice versa through the path of Stensen’s duct, emphasizing the clinical impact of the identification and complete removal of an APG in the treatment of a parotid neoplasm.

The mean size of an APG was 15.8 mm × 5.0 mm on CT in the present study, which corresponds with the “pea-to-kidney bean size” described by Frommer in his cadaveric study from 1977 [1]. Furthermore, we found that the mean distance from the APG to the main gland was 10.5 mm, and ranged from 1–37 mm. This is a relatively longer distance compared with that reported in a cadaveric study, which showed a mean distance of 6 mm between the APG and main gland [1]. The evaluation of a mid-cheek mass can be extremely challenging. Lesions in this area may arise from normal anatomic anterior facial structures including the skin, fat, vessels, lymphatics, and nerves [2, 3, 7]. In addition, an APG neoplasm should be suspected in any patient presenting with a mid-cheek mass. Based on the results of the present study involving 1,600 parotid sides, mid-cheek masses with a separation longer than 37 mm from the main parotid gland could be considered to have not originated from an APG.

In the present study, we found age-related changes in the incidence rates of APGs and FPs. With regard to APGs, the incidence decreased with increasing age, whereas with regard to FPs, the incidence increased with increasing age. These age-related changes in the incidence rates of APGs and FPs showed significant negative and positive linear correlations of r = -0.865 and r = 0.782, respectively. The higher incidence of an APG in younger patients and its decrease with increasing age can be attributed to the fact that an APG is a kind of developmental variation of a premature parotid gland, and as the main parotid gland matures and develops with age, the APG would become fused with the main parotid gland, becoming an FP. Indeed, the weight and volume of the parotid gland usually increase with increasing age; thus an APG can become an FP as the parotid gland grows large enough to be connected with the APG [10, 11]. These increases in parotid weight and volume are attributed to increases in adipose and fibrovascular tissues with age, although the proportion of functional parenchyma in the gland decreases in older people [11, 12]. In fact, in contrast to the APG, the incidence of an FP significantly increased with increasing age. In addition, when we analysed the incidence of an anterior extension of the parotid gland (either an APG or an FP), the incidence rates were similar across all age groups. These findings support the above-mentioned presumption and suggest that APGs and FPs are not completely different variations, but only different types of anterior extensions of the parotid gland. However, it is also possible that the incidence of the APG and FP does not change with age; instead, the ability of CT to detect an APG or FP might vary with patient age, possibly due to alterations in components of the parotid gland, such as the glandular parenchyma, fat, and fibrovascular tissue.

The present study used CT images of normal subjects as the data source. Therefore, the study has some fundamental limitations. The most important limitation is that CT-identified APGs were not biopsied for histological confirmation. Instead, we measured Hounsfield units of suspicious APGs to show that APGs had the same density as the main parotid gland. However, this method could yield some false-positive and false-negative results, especially in the case of small APGs, for which Hounsfield units might not be accurate. Therefore, in clinical settings, additional diagnostic methods such as ultrasound-guided fine-needle aspiration cytology or core-needle biopsy must be used to differentiate a true APG (as a normal variation) form other pathological conditions.

## Conclusion

The present study showed that CT might be helpful in the identification of an anterior extension of the parotid gland including APGs and FPs, with 10.2% and 28.3% incidence rates, respectively. The anatomical information gained from this study contributes to a better understanding of APGs and FPs and how these structures change with age. We hope that our methodological approaches and results will be useful in differential diagnosis of mid-cheek mass and determination of appropriate treatment extent for parotid tumours involving APGs or FPs.

## Supporting information

S1 File Data set of APGs and FPs. (XLSX)
